# Magnet-assisted double-wire technique for patient with difficult endoscopic retrograde cholangiopancreatography cannulation

**DOI:** 10.1055/a-2194-4607

**Published:** 2023-11-21

**Authors:** Hui Gong, Zhiyin Huang, LinJie Guo, Wenjuan Yang, Bing Hu

**Affiliations:** 1 Department of Gastroenterology and Hepatology, West China Hospital, Sichuan University, China, Chengdu, China

A 70-year-old man was referred to our hospital with acute abdominal pain, jaundice, and fever. Magnetic resonance cholangiopancreatography showed a dilated common bile duct obstructed by an 11 × 7 mm fusiform gallstone. Total bilirubin was 38.96 µmol/L. After anti-infection and fluid rehydration, endoscopic retrograde cholangiopancreatography (ERCP) was performed.

ERCP revealed a periampullary diverticulum, with the papilla at its side edge in the 8 o’clock position. Several cannulation attempts failed because the orifice of the papilla was obscured and faced into the diverticulum. For this situation, we secured a ring-shaped magnet, with a short string through the hole, using a clip 1 . Next, a large circular magnet with powerful magnetism was placed on the patient’s external abdominal wall. By slowly moving the external magnet, the internal ring-shaped magnet could be moved to the opposite side of the diverticulum. The orifice was pulled toward the outside of the diverticulum and was stabilized by the magnetic force. Deep biliary papillotomy cannulation was successful using pancreatic guidewire-assisted biliary cannulation with magnet assistance. Finally, the stone in the dilated common bile duct was revealed by cholangiography and cleared after papillary balloon dilation ( Fig. 1 , Video 1 ).

**Fig. 1:**
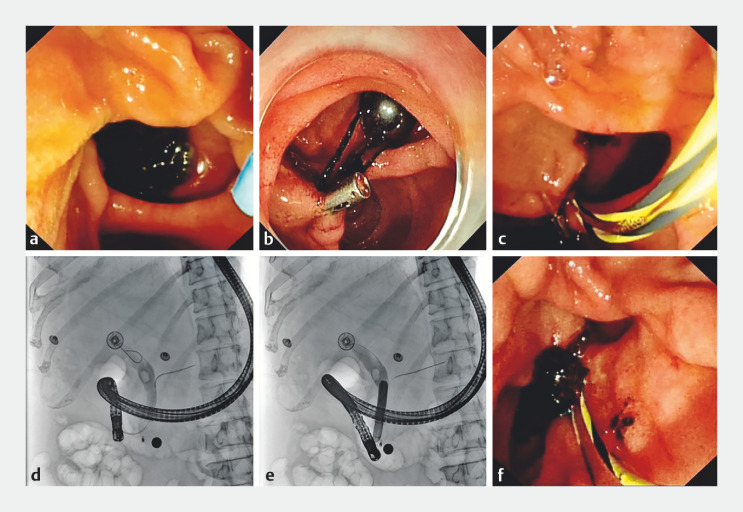
Magnet-assisted double-wire technique in a patient with difficult endoscopic retrograde cholangiopancreatography cannulation. **a** The papilla was hard to view because it was located in the medial wall of the duodenum, and the clip traction effect was not successful. **b** A ring-shaped magnet was attached to the lower edge of the papilla with a metal clip. **c** Following cannulation of the pancreatic duct with a guidewire, a second zebra guidewire was inserted into the bile duct. This double guidewire technique maintained accessibility of the papilla to facilitate successful cannulation. **d** The bile duct stone was revealed by cholangiography. **e** The duodenal papilla was dilated using a dilation balloon. **f** The bile duct stone was removed.

Magnet-assisted double-wire technique for patient with difficult endoscopic retrograde cholangiopancreatography cannulation.Video 1

Ampulla cannulation is the primary and most important step during ERCP. Various methods, such as double-wire technique and precut, have been used to increase the success rate of ERCP cannulation; however, certain cases are still difficult due to the altered anatomical position of the ampulla. In this case, we used the magnet-assisted double-wire technique as a novel traction method that can achieve ERCP success in difficult biliary cannulation.

Endoscopy_UCTN_Code_TTT_1AR_2AC

## Notes

Endoscopy E-Videos https://eref.thieme.de/e-videos
*E-Videos* is an open access online section of the journal *Endoscopy* , reporting on interesting cases and new techniques in gastroenterological endoscopy. All papers include a high-quality video and are published with a Creative Commons CC-BY license. Endoscopy E-Videos qualify for HINARI discounts and waivers and eligibility is automatically checked during the submission process. We grant 100% waivers to articles whose corresponding authors are based in Group A countries and 50% waivers to those who are based in Group B countries as classified by Research4Life (see: https://www.research4life.org/access/eligibility/ ). This section has its own submission website at https://mc.manuscriptcentral.com/e-videos .
